# Judicial diplomacy of the German Federal Constitutional Court: bilateral court meetings as a novel data source to assess transnational communication of constitutional courts

**DOI:** 10.1007/s12286-021-00499-0

**Published:** 2021-12-20

**Authors:** Philipp Meyer

**Affiliations:** Department of Political Science, Leibniz University Hannover, Schneiderberg 50, 30167 Hannover, Germany

**Keywords:** Judicial diplomacy, Transnational judicial communication, Qualitative content analysis, Semantic network analysis, Justizdiplomatie, Transnationale gerichtliche Kommunikation, Qualitative Inhaltsanalyse, Semantische Netzwerkanalyse

## Abstract

Judicial diplomacy describes the courts’ efforts to promote liberal democracy and protect their institutional authority. Bilateral court meetings are essential for judicial diplomacy, encompassing jurisprudential (e.g., discussion of case law) and aims of strategic (e.g., maximising influence). This study presents a novel approach to assess such meetings. It analyses the German Federal Constitutional Court meeting reports between 1998–2019, using content and semantic network analysis. The content analysis shows that court meetings focus on jurisprudential aspects; however, strategic considerations also play a role in discussions with interlocutors from emerging democracies. These findings are substantiated by the semantic network analysis, which discloses that recent case law, Europeanisation, and globalisation are the main issues discussed. Hence, this study presents an analysis of a novel data source. Further, it contributes to judicial politics research as transnational court meetings could be a missing link to understand legal citation networks.

**Supplementary Information:**

The online version of this article (10.1007/s12286-021-00499-0) contains supplementary material, which is available to authorized users.

## Abstract (de)

Verfassungsgerichte versuchen aktiv, mittels diplomatischer Bemühungen, liberale demokratische Strukturen zu fördern und gleichzeitig ihre institutionelle Autorität zu stärken. Ein wesentliches Element dieser Gerichtsdiplomatie sind bilaterale Treffen, mit welchen nationale Gerichte sowohl juristische (z. B. mittels Fachgespräche über nationale Rechtssprechungspraktiken) als auch strategische (z. B. Maximierung des eigenen innenpolitischen Einflusses) Aspekte diskutieren. Mittels einer Analyse von Pressemitteilungen bezüglich bilateraler Treffen des deutschen Bundesverfassungsgerichts zwischen 1998–2019, stellt diese Studie einen neuen Ansatz zur Untersuchung von bilateralen Treffen vor. Methodisch werden sowohl eine Inhalts- als auch eine semantische Netzwerkanalyse durchgeführt. Die Inhaltsanalyse zeigt, dass die bilateralen Treffen auf rechtswissenschaftliche Aspekte fokussiert sind, während strategische Überlegungen insbesondere bei Diskussionen mit Gesprächspartnern aus sich in Entwicklung befindlichen Demokratien eine Rolle spielen. Diese Ergebnisse werden durch die semantische Netzwerkanalyse bestätigt, die offenlegt, dass die Hauptthemen, die diskutiert werden, nationale Rechtssprechungspraktiken, Europäisierung und Globalisierung sind. Diese Studie präsentiert eine Analyse einer neuartigen Datenquelle. Darüber hinaus leistet die Studie einen Beitrag zur Gerichtsforschung, indem sie zeigt, dass transnationale Gerichtstreffen dabei helfen können juristische Zitationsnetzwerke und transnationale Entscheidungsverweise zu verstehen.

## Introduction

The concept of judicialisation refers to the “global expansion of judicial power” (Tate and Vallinder 1995) and to the increasing influence of national judges which “intervene in legislative processes, establishing limits on law-making behavior, reconfiguring policy-making environments, even drafting the precise terms of legislation” (Stone Sweet 2000, p. 1). However, several scholars argue that due to legal globalisation processes^1^, national judiciaries’ regulatory scope steadily shrinks, as domestic policies are becoming determined by supranational and international regulations and institutions (Benvenisti and Downs 2009; Jupille and Caporaso 2009; Herschinger et al. 2011). In order to encounter this potential loss of power, domestic courts are found to be engaged in judicial diplomacy to promote global justice, to protect the domestic rule of law and maximise their influence both internationally and nationally (Slaughter 2004; Benvenisti and Downs 2009; Law 2015; Davies 2020).

Judicial diplomacy encompasses two aspects. First, dialogue means “communication among open-minded peers for the sake of mutual learning and reasoned problem-solving” (Law 2015, p. 1023). Second, the “exercise in power politics” (Law 2015, p. 1023), as national courts compete for influence, authority, and prestige (Slaughter 2004; Garoupa and Ginsburg 2015; Davies 2020). Courts engage in diplomatic efforts to build and increase international credibility and authority, which they then re-import into their respective national system (Claes and de Visser 2012). Diplomacy seems to have become a viable strategy for national courts, to the extent that some judges have already described themselves “as ‘judicial statesperson[s]’ or ‘ambassadors’ with responsibility for representing their court and its jurisprudence abroad” (Davies 2020, p. 78).

Besides institutionalised networks, like the *European Judicial Network*, the *Conference of European National Courts*, or the *International Association of Women Judges*, which have already been analysed in prior studies (Slaughter 2004; Claes and de Visser 2012; de Visser and Claes 2013), bilateral court meetings are also an essential aspect of judicial diplomacy. For example, the Supreme Court of Canada meets about 25 foreign delegations every year (Mak 2013). Law (2015) and Davies (2020) show that the Supreme Courts of Hong Kong, Taiwan, South Korea, the United States, and the UK regularly met with foreign courts. In the following, this study will focus on bilateral court meetings, as they have, compared with judicial networks, the advantage for judges to advocate and discuss particular approaches to legal issues in greater detail (Claes and de Visser 2012; Mak 2013). More profound knowledge of what such meetings are about will contribute to a better understanding of how the global judicial community works (on a global legal society see, e.g. Slaughter 2004).

However, existing studies on judicial diplomacy lack rigorous methods, systematic analyses, and generalisable results. For example, Slaughter (2004, p. 261) states that her results are “anecdotal, though numerous” and that “[m]ore systematic research is required.” Moreover, the analysis by Davies (2020) is based on only eight interviews, the studies by de Visser and Claes (2012, 2013) remain at the level of descriptions of existing judicial networks in Europe, and Law (2015) presents in-depth descriptions of the diplomacy efforts by five Supreme Courts in Asia. In this context, Meierheinrich (2009, p. 85) concludes that the existing studies lack “a proper attention to research design, i.e. the rules of social inquiry.”

This study asks what aspects are associated with bilateral meetings. An answer will be presented using a novel data source—reports on transnational bilateral meetings^2^ published by the German Federal Constitutional Court (FCC) between 1998 to 2019—and applying two different research methods—content and semantic network analysis. During the 21 years under scrutiny, the German court has participated in 137 meetings. As such, this study represents the first approach for a systematic, large-scale analysis of international court relations.

The German case is particularly well suited for an empirical assessment of bilateral meetings for several reasons. First, the FCC is among the most powerful and influential courts worldwide (Kommers 1994). Its decisions affect Germany’s political and legal systems and the European Union alike (Dyevre 2011). In conjunction with its strong legal and political authority (Krehbiel 2019), these aspects have mainly contributed to mirroring its institutional design by high courts worldwide (Navia and Ríos-Figueroa 2005; Hönnige 2007). Moreover, the FCC’s case law, institutional setting, and internal procedures are in the interest of several national high courts, as evidenced by the fact that the German court is one of the most frequently referenced and considered courts worldwide (Law 2015). This implies that the results of this study produce insights into the dynamics of judicial diplomacy that can travel to similarly important high courts like the French *Conseil constitutionnel*, the US Supreme Court, and even supranational courts like the European Court of Human Rights (similar considerations can be found in Law 2015).

This study is structured in three steps. First, the existing literature on judicial diplomacy and judicial behaviour will be reviewed to show the heterogeneous nature of the existing concepts and to identify central aspects of bilateral meetings. Second, a directed content analysis of the FCC’s meeting reports will be presented based on a coding scheme. The results offer supporting evidence for the aspects that have been theoretically associated with bilateral meetings. Finally, a semantic network analysis based on the full text of the meeting reports is presented to validate the content analysis by displaying the significant issues discussed by the FCC and its interlocutors.

The presented results show that bilateral meetings primarily focused on jurisprudential aspects, especially to promote the rule of law, to discuss case law, and to examine specific aspects of judicial decision-making. Nevertheless, it is also found that the FCC and its judges use the meetings from a strategic perspective to discuss issues like Europeanization, judicial independence, and democratisation. The semantic network analysis validates the content analysis results by displaying that the issues discussed at these meetings cluster around themes like case law, Europe, globalisation, basic rights, and judicial independence.

This study contributes to the existing literature, as it presents both a novel data source and a multi-methods approach for the analysis of transjudicial interactions. Furthermore, it shows that courts discuss and share national case law developments in the context of bilateral meetings. Transnational meetings allow courts to facilitate understanding of their case law, “which in turn can encourage the citation of their decisions by other courts outside of their jurisdiction” (Davies 2020, p. 82). Hence, the results presented here will be beneficial for the judicial politics literature and its increasing interest in citation networks and the citation of foreign precedents (Fowler et al. 2007; Dyevre 2010; Dyevre et al. 2019), as bilateral court meetings could be a missing link for understanding the global judicial architecture.

## Judicial diplomacy: conceptualisations, aims, and incentives

This study is about bilateral court meetings as elements of judicial diplomacy. As such, it is not about mutual decision citations. Thereby, it follows the argumentation by Meierheinrich (2009) and Law and Chang (2011) that one-sided citations are more a form of hierarchy than a two-way dialogue. Nevertheless, court meetings can facilitate citations, as participating judges potentially can be persuaded of the value of foreign decisions “in helping him or her sort through a knotty legal problem” (Slaughter 2004, p. 101). Citations are, therefore, a possible outcome of bilateral meetings rather than their expression. Further, this study is also not about judicial networks, as they are found to be thematically too broad “for a fruitful exchange of legal ideas” (Mak 2013, p. 85), while bilateral meetings provide the opportunity to discuss specific issues.

All varieties of judicial diplomacy—be it networks, seminars, or meetings—are assumed to foster the creation of a globalised epistemic judicial community to promote the rule of law, judicial independence, and the judiciaries’ role as an essential constitutional branch. Scholars have found that international relations between courts foster the mutual understanding of legal systems and specific national constitutional requirements, which facilitates not only international cooperation but also strengthen national courts to defend their independence and to protect liberal democracy (Slaughter 2004; Mak 2013; de Visser and Claes 2013; Law 2015; Dressel et al. 2017). Overall, judicial diplomacy is argued to be based on courts “mutual (self-)perceptions as belonging to the same trans-national judicial community”, providing them with an opportunity “to share and discuss common problems, to learn from foreign experiences and to tackle common challenges” (de Visser and Claes 2013, p. 347).

Compared to this general understanding of judicial diplomacy, existing conceptualisations are rather heterogeneous. Martinez (2003) argues that transnational interactions are context-related and should be conceptualised either as *international court-national court relationships (Type 2)* or as *national court-national court relationships (Type 3)*. For each type, she presents a different set of relevant issues. For example, Type 2 relationships primarily involve the separation of power, national sovereignty, and institutional competencies, while Type 3 relationships are occupied with issues like national sovereignty, the interdependence of legal systems, and institutional competencies. Benvenisti and Downs (2009) present the concept of *interjudicial cooperation*; however, they do not offer a clear definition apart from the notion that it is about a mutual exchange of information.

De Visser and Claes (2013) focus on *judicial networks*. They argue that networks are based on judicial communities that share beliefs, values, and a basic understanding of legal systems. Based on descriptions of several European networks, de Visser and Claes (2013, p. 366) conclude that judicial networks link “national courts with one another. In addition, they can contribute to improving the interaction between the European Courts and their national counterparts”. In an earlier study, Claes and de Visser (2012) approached *judicial networks* by discussing three central concepts: network, dialogue (dialogue-as-conversation and dialogue-as-deliberation), and constitutional pluralism. In their view, courts are the guardians of the legal order, and judicial networks help them increase their bi- and multilateral relationships to strengthen their role as neutral arbiters. Slaughter (2004) distinguishes between three types of judicial networks: 1) *information networks*, focusing on transjudicial communication and bilateral discussion, 2) *enforcement networks*, focusing on “enhancing cooperation among national regulators to enforce existing national laws and rules. As the subjects they regulate—from criminals to corporations—move across borders, they must expand their regulatory reach by initiating contact with their foreign counterparts” (Slaughter 2004, p. 55), 3) *harmonisation networks*, aiming to facilitate market harmonisation negotiations.^3^

Based on interviews with judges from several national courts, Mak (2013) assesses *international judicial relations*. Her analysis covers institutionalised networks as well as bilateral meetings, and she concludes that although judges “appreciate the possibilities for exchange with their foreign colleagues”, the actual benefit of institutionalised networks is somewhat limited due to their broad agenda. In contrast, bilateral court meetings are found to “provide a setting for deliberations, rather than mere conversation” (Mak 2013, p. 112). Similarly, Davies (2020) understands *judicial diplomacy* as regular interactions between judges from national and supranational courts. Davies focused on judges’ aims when engaging in judicial diplomacy based on eight interviews with judges from the UK Supreme Court. He concludes that judges “look to improve the quality of decision-making […] [and to maintain] good inter-institutional relations with supranational judges and maximising their influence at the supranational level” (Davies 2020, p. 94). One possible explanation of the results by Davies is that institutionalised events like bilateral meetings could be the starting point of more intense informal relationships (Dressel et al. 2017), as judges recognise the benefits of transnational interactions such as a steady flow of information, peer support, and also protection. This means that the effectiveness of judicial diplomacy is connected to the creation of informal between-bench-ties in the wake of formal diplomatic efforts such as meetings and networks (Dressel et al. 2017) since they provide the necessary arena to discuss specific issues and encourage judges to apply and cite foreign precedents in order to challenge domestic political interests (Mak 2013; Dressel et al. 2017; Davies 2020).

Dressel et al. (2017, 2018) assessed this informal dimension of judicial politics by establishing the concepts of *on-bench, off-bench*, and *between-bench relations*. The first two focus on internal processes within national judiciaries; only the latter will be discussed here. Although the authors exclude “the formal dimension of […] transnational organisations like the European Judicial Network” from their concept of *between-bench relations*, they explicitly recognise that from institutionalised forms of judicial diplomacy “often informal structures and relationships [between judges emerge]” (Dressel et al. 2017, p. 419). Accordingly, this “encourage judges to make decisions that might challenge domestic political interest” (Dressel et al. 2017, p. 423). In this regard, transnational between-bench relations “are important not only for transmitting ideas and technical knowledge but also for protecting judicial autonomy and fostering more assertive behavior against other political branches.” As such, they may be “critical to the dynamics surrounding judicial independence and autonomy because they provide much-needed support for one of the weakest branches of government” (Dressel et al. 2017, pp. 423–424). Hence, transnational judicial relations empower judges by providing them with new ideas, information about foreign case law, peer-support, and an arena to increase the institutional standing of the court they represent. Similar conclusions are presented by Garoupa and Ginsburg (2015) in their discussion on the benefits of transjudicial cooperation for national courts to increase their global reputation.

In summary, transnational judicial interactions have already received scholarly attention, and it is argued that they are a catalyst for creating, implementing, and maintaining global judicial communication. Transnational bilateral court meetings are understood as “horizontal relations across national borders” (Slaughter 2004, p. 69) to foster dialogue about case law, the rule of law, and judicial independence. Scholars have found that meetings are opportunities for national judges to exchange information and ideas and to build transnational relationships (Dressel et al. 2017), while they also present a viable arena for national courts to strengthen their authority, safeguard domestic democratic processes and increase their reputation both globally and nationally (Slaughter 2004; Benvenisti and Downs 2009; Mak 2013; Garoupa and Ginsburg 2015).

### Incentives and aims of bilateral court meetings

Judicial politics scholars model judicial behaviour mainly based on the legal, the attitudinal, and the strategic models. While the legal model assumes that judges follow the rules and regulations and solely apply the law as a neutral arbiter, and the attitudinal model assumes that judges decide cases based on their preferences and being policy-seeking actors (see, e.g., Segal and Spaeth 2002, pp. 48–112; Baum 2008, pp. 1–24), the strategic model takes up a much broader perspective. It treats judges and courts as strategic political actors, considering that they are legal professionals and policy-seekers. Moreover, the model assumes that judges’ actions correspond to other actors’ preferences and actions and that they are aware of the institutional context they operate in (see, e.g., Epstein and Knight 1998; Carrubba 2003; Staton 2010; Engst 2021). As international law and regulations increasingly narrow the judicial scope of national judiciaries, strategic courts are in dire need of a strategy “for both protecting their authority and safeguarding the domestic democratic processes” (Benvenisti and Downs 2009, p. 65). As argued above, bilateral court meetings and international relations are valuable for courts and their judges to achieve both.

Scholars have identified two incentives judges associate with bilateral meetings. First, there are *practical incentives*. Since constitutional law has become increasingly globalised (Slaughter 2004; Tushnet 2019), judges need to understand other national legal systems and foreign case law. Claes and de Visser (2012, p. 111) argue that “[t]he internationalised nature of litigation makes knowledge about other legal systems a prerequisite to being able to dispense justice in an individual case.” Similarly, Garoupa and Ginsburg (2015, p. 170) argue that “globalisation increases the probability that courts in different contexts will indeed face common issues, and a natural response is to see how other courts have handled similar questions.” In this regard, bilateral meetings are perceived to be perfect opportunities for judges to understand and learn from each other (Benvenisti and Downs 2009; Mak 2013; Garoupa and Ginsburg 2015), which also can emerge in strong informal structures and relationships that affect national case law due to knowledge transfer and peer-support (Dressel et al. 2017) Second, there are *authoritative incentives*. Since the demand for global judicial reputation has increased due to the stated judicial globalisation (Garoupa and Ginsburg 2015), bilateral meetings provide “an effective check on executive power at the national and international levels alike and promoting ideas of the rule of law in the global sphere” (Benvenisti and Downs 2009, p. 60). As such, bilateral meetings can foster a court’s authority and legitimacy (Garoupa and Ginsburg 2015), as it can use these meetings “to build credibility and authority outside [its own] legal system, which is subsequently imported back into that national system” (Claes and de Visser 2012, pp. 111–112).

Additionally to these incentives, scholars have identified the aims individual judges associate with bilateral meetings. Davies (2020), for example, presents two aims: 1) *jurisprudential* and 2) *strategic*.^4^ The first relates to the decision-making practices and the development of legal principles on the domestic and the international level. In this context, Bilateral meetings are assumed to be an arena for discussions about 1) recent cases and the quality of legal reasoning (Claes and de Visser 2012); 2) the development of national case law and procedural issues (Davies 2020); 3) aspects of court administration (Mak 2013). Moreover, meetings are opportunities to understand foreign decision-making. Hence, with bilateral meetings, judges and courts aim to 1) improve the quality of domestic decision-making by exchanging views of the application of legal principles and discussing individual cases and recent developments in national case law and 2) to foster mutual knowledge and understanding of different procedural issues and domestic court administration. Ginsburg (2008) has argued that the diffusion of constitutional ideas is more likely between countries with similar characteristics, and the legal tradition is one of these characteristics. Hence, this study hypotheses that jurisprudential aims are more relevant in meetings between courts that share commonalities, such as legal tradition or institutional heritage.

On the contrary, the strategic aim is concerned with courts’ authority, legitimacy, reputation, and political influence. In this vein, judges use bilateral meetings to discuss substantive matters beyond national borders and promote the rule of law, liberal democracy, and judicial independence (de Visser and Claes 2013; Garoupa and Ginsburg 2015; Davies 2020). Hence, from a strategic point of view, judges and courts participate in bilateral meetings to 1) maximise international influence by promoting national decision-making practices, 2) strengthen the domestic position by increasing international prestige, and 3) promote the rule of law and judicial independence. Martinez (2003) has argued that especially the relationships between international and national judicial actors are focused on the separation of power, national sovereignty and political influence and competencies, while other scholars have argued that the promotion of democracy is a central aim in meetings with courts from non-democratic states or even when meeting courts from emerging democracies (Davies 2020). Hence, this study hypotheses that strategic aims are more relevant in meetings between national courts and international courts or international organisations.

## Research design

According to Meierheinrich (2009, p. 85), existing research on judicial diplomacy is either anecdotal or descriptive and is missing “a proper attention to research design”. This shortcoming will be addressed by this study focusing on one particular case, the German Federal Constitutional Court (FCC), and presents a novel data source, reports on meetings from and to the FCC, to test the formulated hypotheses empirically. Thereby, this study presents a systematic and data-driven approach to assess judicial diplomacy.

The study will proceed in the following steps. First, a brief discussion shows why the German Federal Constitutional Court is suitable for analysing bilateral court meetings. Second, the data will be presented. Finally, two methods—qualitative content analysis and semantic network analysis—will be discussed to show their suitability to answer the research question.

### The case: the German Federal Constitutional Court

The highest courts around the world put effort into judicial diplomacy. Table 1 lists all constitutional courts of EU-member states. The table shows whether a court has a dedicated section on its website concerning international relations, how it is labelled, and if reports on bilateral meetings are published.

**Table 1:** Information on judicial diplomacy on websites of constitutional courts in EU-member states

Country	Apex court	Dedicated website section	Website section name	Meeting reports published online
Argentina	*Supreme Court of Argentina*	No	/	No
Australia	*High Court of Australia*	No	/	No
Brazil	*Federal Supreme Court*	Yes	International cooperation	No
Canada	*Supreme Court of Canada*	No	/	No
Colombia	*Constitutional Court of Colombia*	No	/	No
India	*Supreme Court of India*	No	/	No
Israel	*Supreme Court of Israel*	No	/	Yes
Japan	*Supreme Court of Japan*	No	/	No
Mongolia	*Constitutional Court of Mongolia*	Yes	International relations	Yes
Niger	*Constitutional council of Niger*	No	/	No
Nigeria	*Supreme Court of Nigeria*	No	/	No
Norway	*Supreme Court of Norway*	Somehow (part of annual report)	The Supreme Court and the international legal community	Somehow (part of annual report)
Pakistan	*Supreme Court of Pakistan*	Somehow (part of annual report)	Foreign tours of the Chief Justice and Judges	Somehow (part of annual report)
Philippines	*Supreme Court of the Philippines*	No	/	Yes
Ruanda	*Supreme Court of Ruanda*	No	/	No
Russia	*Constitutional Court of the Russian Federation*	Yes	International relations	Yes
South Africa	*Constitutional Court of South Africa*	No	/	No
South Korea	*Supreme Court of Korea*	Yes	International relations	Yes
Turkey	*Constitutional Court of the Republic of Turkey*	Yes	International relations	Somehow (part of annual report)
United Kingdom	*High Court of Justice*	Yes	International judicial relations	No
United States	*Supreme Court of the United States*	No	/	No

Information extracted from the courts websites (last access of all websites: 29 October 2020)

The tables depict that some courts do publish reports on bilateral meetings and have dedicated international relations sections (e.g., Germany, Latvia), some only publish meeting reports (e.g., Belgium, Romania), some have a section on their website but do not publish information on activities (e.g., Italy), and others list neither of both (e.g., Poland, Bulgaria). Besides, some courts publish information on their international activities in their annual reports (e.g., France). Interestingly, the labelling of the websites shows a rather heterogeneous picture, similar to the above-reviewed scholarly concepts.

The table shows that judicial diplomacy is a common phenomenon in Europe. Aside from this commonality, several more aspects justify the German Federal Constitutional Court (FCC) as a suitable case selection for an empirical examination of bilateral court meetings. First, since its establishment, the FCC has developed a crisis-resistant high level of public support (Schaal 2015) and a solid legal and political authority that extends far beyond the German political system (Dyevre 2011; Krehbiel 2019). These aspects have motivated constitutional assemblies to adapt or mirror the institutional design of the German court when establishing judicial review, as it was shown for the newly established courts in post-communist countries (Hönnige 2007; Holtz-Bacha 2017) as well as for judiciaries in Latin America (Navia and Ríos-Figueroa 2005). Finally, the FCC’s public relations approach shares central aspects with several other highest courts’ communication strategies worldwide (Davis and Taras 2017; Meyer 2020, 2021a, b).

Second, the studies by Mak (2013), Meierheinrich (2009), and Davies (2020) highlight the importance of the legal tradition to understand transnational judicial communication. Legal traditions define the way how legal systems are structured, as they are characterised by a “set of deeply rooted, historically conditioned attitudes about the nature of law, about the role of law in the society and the polity, about the proper organisation and operation of a legal system, and about the way law is or should be made [and] applied” (Tetley 2000, p. 682). Two legal traditions are most relevant: civil law, for example, the German legal system, and common law, from which, most prominently, the legal systems in the UK or the US draw their origins. The civil law tradition is based on legislation with written codified constitutions. These documents manage civil and political life; however, they are not just “a list of special rules for particular situations; rather, a body of general principles carefully arranged and closely integrated” (Dainow 1966, p. 424). The civil law tradition distinguishes between constitutional law and ordinary laws. Most European countries and several states in Africa and Asia—especially Taiwan, South Korea, and Japan (Law 2015)—have adopted this tradition. Common law is based on case law, which means that if “a court decided a particular case, its decision was not only the law for those parties, but had to be followed in future cases of the same sort, thereby becoming a part of the general common law. This, the common law, as a body of law, consisted of all the rules that could be generalised out of judicial decisions” (Dainow 1966, pp. 424–425). The common law tradition is dominant among (former) Commonwealth countries. In conclusion, civil law rests on codified, structured declarations summarising broad and abstract principles, while in common law, regulations are mostly uncodified, and legality is based on specific cases making common law more detailed and concrete (Tetley 2000).^5^ Through its role model character as a specialised court within the civil law tradition, the German Federal Constitutional Court is found to be not only one of the most referenced court worldwide (Law and Chang 2011; Law 2015)^6^ but also a valued and highly demanded dialogue partner (Mak 2013). However, this holds particularly true for other civil law courts as a “shared ideological background, reflecting the principle of the rule of law, and a shared level of legal development form a basis for further exchange with courts” (Mak 2013, p. 94).

These two aspects—being both an institutional blueprint and a reference point for other highest courts—imply that the FCC presents an empirically important case that is representative of others, which is why it serves as an appropriate starting point for understanding a larger—albeit similar—set of cases.

### The data: meeting reports published by the German Federal Constitutional Court

This study relies on a novel data source: official meeting reports published by the German Federal Constitutional Court between 1998 and 2019. Existing research mainly uses interviews with judges (Mak 2013; Davies 2020) or descriptions of empirical processes (Slaughter 2004). This study presents a first attempt to assess judicial diplomacy efforts with a quantitative approach.

The meeting reports analysed in this study are written and published by the court’s public relations office, headed by a trained judge, who is selected for a two to three year period by the FCC’s president (Meyer 2020). The public relations office was established in 1996 in the wake of an institutional crisis caused by mishandled communication of two significant cases (Schaal 2015; Holtz-Bacha 2017). The majority of press releases the Court publishes are comprehensive summaries of significant decisions. The press releases are disseminated via an E‑Mail newsletter, Twitter, and on the FCC’s website. Moreover, the FCC also uses press releases to announce upcoming decisions and oral hearings (Meyer 2020).

The meeting reports are either about meetings between judges of the FCC and national political actors (e.g., members of the Bundestag or the German federal chancellor) or about meetings between judges and foreign actors like judges from other national highest courts and supranational courts (e.g., the Court of Justice of the European Union), delegations from European institutions (e.g., the European Commission), foreign political actors (e.g., the US Consul General in Germany), and also from foreign non-governmental actors (e.g., the Association of Lawyers of Russia). The meetings were held either during visits from delegations to the Court or during visits from the FCC to foreign courts or political institutions. As this study is focused on judicial diplomacy, it only assesses the reports on meetings with foreign actors and leaves meetings with domestic actors aside.

The full text of the meeting reports was scraped from the FCC’s website. The first meeting report published dates back to February 1998, which is why this year was chosen as the analysis’s starting point. The year 2019 was selected as the endpoint, as most meetings planned for 2020 were postponed due to contact restrictions in the wake of the COVID-19 pandemic.^7^ Therefore, a meeting with the Slovak Constitutional Court delegation in December 2019 is the last data entry. Overall, 121 meeting reports were scraped from the Court’s websites. However, as some reports cover several meetings in the context of judges’ trips to different countries, the data contains 137 transnational meetings in total.

The FCC’s meeting reports are texts that are not longer than one page, covering the following sections/information: 1) date of the meeting, name of the visiting or visited actor, country, and the names of the participants; 2) issues discussed at the meeting, and occasionally 3) additional events after the initial meeting (e.g., a ceremonial act or dinner with government officials). Hence, the reports are brief summaries of who met, when and on what occasion, and what topics were discussed.^8^ The issues-discussed and the nature of the meeting (e.g., ceremonial, professional discussion) are either briefly mentioned or explained in one to two sentences. In this respect, the reports cannot and do not want to be a complete summary of the meetings; however, they seem to represent standard meeting report conventions.^9^ Overall, because of these reports’ rather technical and result-focused style, they are in line with the established perception of court communication as neutral, objective, and open to external scrutiny (Meyer 2021b). In this regard, Johnston (2018, p. 530) has argued that court press releases are a public-interest communication with which a court is more of a “facilitator and enabler of [transparency and openness] rather than [an institution] that pro-actively seeks to manage a message.” Hence, although these reports represent self-representations of the FCC, it is suitable to assume that they deliver rather objective descriptions of the meetings. As such, these reports provide a suitable and novel data source to assess judicial diplomacy. Examples of meeting reports are shown in Appendix A (available as online supplementary material).

### Methods

This study applies a multiple methods approach. First, qualitative content analysis will be executed to offer supporting evidence for the two hypotheses. Second, a semantic network analysis based on the full texts shows which issues are discussed at bilateral meetings over time. The network analysis intends to strengthen the initial results from the qualitative content analysis.

Qualitative content analysis is a reasonable method choice, considering this study’s aim—finding support for bilateral meetings’ *strategic *and *jurisprudential *aims—and the novel data source used here. In contrast to quantitative content analysis, which focuses on the manifest content (e.g., the number of times a specific word, phrase, or the like occurs in a text) of texts (Grimmer and Stewart 2013), the qualitative content analysis assesses latent textual aspects, thereby, examining language to classify text into meaningful categories. In other words, “qualitative content analysis is defined as a research method for the subjective interpretation of the content of text data through the systematic classification process of coding and identifying themes or patterns” (Hsieh and Shannon 2005, p. 1278).

This study conducts a *directed content analysis*, which attempts to validate or extend a theoretical framework by identifying specific structures within texts (Jauch et al. 1980; Hsieh and Shannon 2005; Mayring 2010). The method is conducted in three steps. First, based on prior studies and existing theories, key concepts are identified as coding categories. Second, for each category, an operational definition is formulated. Third, the texts are coded according to the defined categories (Hsieh and Shannon 2005; Mayring 2010). Directed content analysis can support or even extend existing theories. The major downsides are, first, a potential bias that will lead researchers to “find evidence that is supportive rather than nonsupportive of a theory” and second, “an overemphasis on the theory can blind researchers to contextual aspects of the phenomenon” (Hsieh and Shannon 2005, p. 1283).

A semantic network analysis based on the full texts of the meeting reports will be conducted to overcome such problems. By utilising algorithms to calculate the proximity of words in text data and relying on social network analysis and graph theory, semantic network analysis yields a quantitative measurement of texts apart from their concrete meaning. This measurement is then converted and illustrated in a network graph, which helps understand the texts’ semantic structures. In short, “[s]emantic networks allow to model semantic relationships that are represented in a graph with labeled nodes and edges” (Drieger 2013, p. 4). These networks help discover qualitative aspects from text and support “processes which involve analytical reasoning and knowledge building” (Drieger 2013, p. 4). In contrast to other automated text analysis methods, semantic network analysis has the advantage to explore the structures and connections between texts and assessing “how words are interconnected and contextually situated in a network structure” (Drieger 2013, p. 15). Due to these advantages, semantic networks are suitable for supplementing the results from the qualitative analysis.

Both methods require preparatory work. The qualitative content analysis necessitates theory-driven coding categories and operational definitions, both derived from the reviewed concepts. Accordingly, following the proposed steps of directed content analysis (Hsieh and Shannon 2005; Mayring 2010), each meeting report was examined and coded in the light of the theoretically assumed *aims* associated with judicial diplomacy: *jurisprudential* and *strategic* Coding categories, definitions, and examples for each category are shown in Appendix B (available as online supplementary material).

The semantic network analysis uses the full texts of the meeting reports. To produce a meaningful semantic network, “a series of preprocessing steps [is necessary] to reduce the awe inspiring diversity of language to a manageable set of features” (Grimmer and Stewart 2013, p. 272). In the first step, a corpus is created by segmenting the texts into word-tokens. Subsequently, punctuations, numbers, capitalisations, stop words, and common words (e.g., *Bundesverfassungsgericht* (Federal Constitutional Court), *Richter* (judges), *Karlsruhe* (the city in which the FCC is located), or the names of the judges) are discarded from the corpus. Additionally, the judges’, institutions’, and states’ names and meeting locations were also erased from the texts. These steps ensure that the remaining word-tokens represent the meetings’ most significant and meaningful words. Finally, this corpus is used to create a *feature co-occurrence matrix* (FCM) that estimates the semantic proximity of each token, which is why the relatively short meeting reports are a suitable data source (Watanabe 2020). In general, an FCM represents a network based on co-occurrences of specific features (in this study tokens) in a defined context (here: all meeting reports).^10^ As a result, a matrix is created that measures the co-occurrences of tokens within all court meeting reports and, thus, represents a semantic network that maps out how the central themes and issues in the meetings are connected and clustered.

### Additional information extracted from the reports

Several additional informational aspects of value for the analysis are extracted from the full text or deduced from data sources like the CIA World Factbook (variable names used in the analysis are depicted in *italics*)*.* First, each meeting report covers the issues discussed during the meeting. As the reports mention the discussed issues in a standard sequence, it is possible to code the *first topic* consistently. The nine identified topics, descriptions, and examples are shown in Appendix C (available as online supplementary material).

Second, the FCC labels its meetings with different *meeting type*s. In its reports, the Court uses several labels like professional discussion (*Fachgespräch*), informational meeting (*Informationsaustausch*), and workshop (*Arbeitssitzung*). Besides, on some occasions, the FCC judges have made representational meetings with representatives of foreign states without participating in professional discussions and workshops (e.g., a visit to Russia to celebrate the 15th anniversary of the Constitution of the Russian Federation). Moreover, some meetings have been combined with additional festivities like award ceremonies, dinners, and lectures, where the judges have met with political actors like city majors, parliamentarians, and even heads of state.

Third, the *legal tradition* of the FCC’s counterpart is also covered. According to the CIA World Factbook (CIA 2020), each meeting partner’s legal heritage was coded, differentiating between civil law, common law, and mixed law tradition. The tradition of supranational meeting partners like the Court of Justice of the European Union, the Annual Meeting of South American Judges, or the African Court on Human and Peoples’ Rights were coded as mixed law since supranational actors are found to draw inspiration from different legal traditions (Nicola 2016).

Fourth, as the FCC has participated in meetings with national highest courts, supranational courts, and national and supranational political actors, the variable *type of meeting partner *displays the FCC’s interlocutor. Finally, scholars argue that democratic courts use bilateral meetings to promote the rule of law and support democracy, especially when meeting with actors from non-democratic states or emerging democracies (Mak 2013; Davies 2020). Thus, the variable *liberal democracy index* measures the democratic development of the FCC’s meeting partner at the meeting time. Information on this variable is obtained from the V‑Dem liberal democracy index, one of the most detailed and comprehensive data sources for comparative country data (Pemstein *et al*. 2020).

## Results

**Fig. 1:**
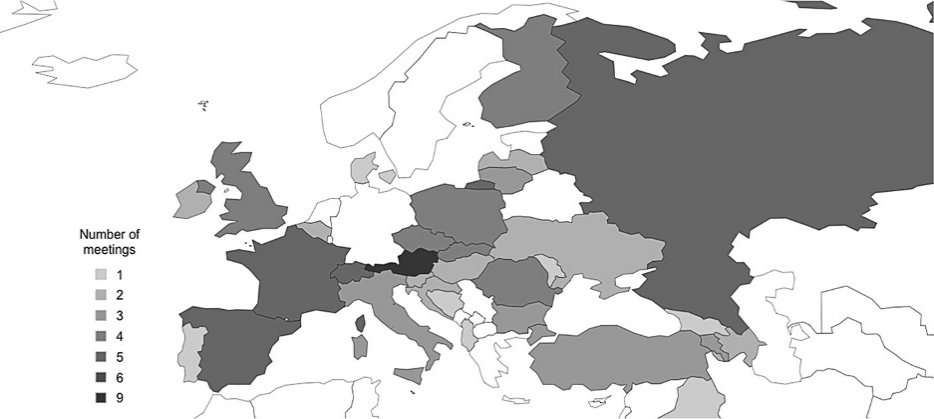
Frequency of FCC meetings with delegations from European countries, 1998–2019

**Fig. 2:**
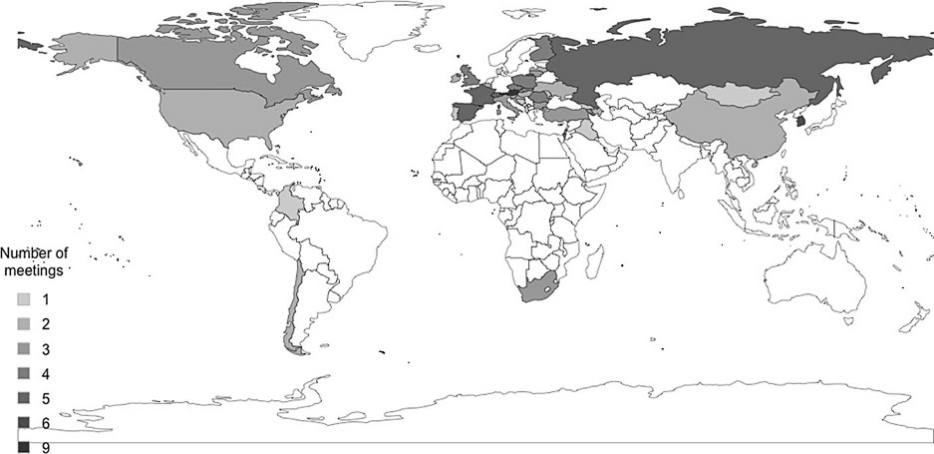
Frequency of FCC meetings with delegations from countries worldwide, 1998–2019

Between 1998 and 2019, the German Federal Constitutional Court has participated in 137 meetings. The most bilateral meetings have taken place in the years 2013 (12), 2012 (11), and 2019 (11), while the fewest meetings took place in 2005 (2), 1999 (1), and 2004 (1). Figs. 1 and 2 illustrate a world map, respectively, a map of Europe, showing the meetings’ frequency by country. Both maps display that the interlocutors mainly came from countries in Europe and Asia. For example, the FCC has met eight times with judges from the Constitutional Court in Austria, six times with judges from the Supreme Court of South Korea, and participated in five meetings with delegations from Russia. Countries in Africa, except for South Africa, have not been meeting partners, which is striking because several African countries have established specialised constitutional courts in recent years (Stroh and Heyl 2015). Meetings with actors from the two Americans are somewhat in-between, with a stronger emphasis on Canada and the United States, not surprising due to their international reputation (Slaughter 2004; Mak 2013; Law 2015).

Nevertheless, both maps only display meetings with delegations from single countries. Thus, they do not capture those with delegations from supranational bodies. Table 2 lists all actors the FCC has met with, the frequency, and the legal tradition. In total, the FCC has met with 57 interlocutors. The majority of meetings were with other national courts (81.75%). The remaining meetings differentiate into those with supranational courts (10.22%), national political actors (2.92%), supranational political actors (2.19%), national legal actors (2.19%), and supranational legal actors (0.73%). Although there are no comprehensive measurements regarding bilateral court meetings of other courts, these numbers seem reasonable when compared with the results presented by Davies (2020), Law (2015), Mak (2013), or compared with annual reports, for example, by the French *Conseil constitutionnel* or the Norwegian Supreme Court.^11^ Finally, the Court has participated in 99 meetings (72.3%) with actors with a civil law tradition, 11 meetings (8%) with actors with a common law tradition, and 27 meetings (19.7%) with actors with a mixed law tradition, which include 17 meetings with supranational actors (see also Table 3). These numbers provide the first evidence that courts more likely meet with actors that share the same legal tradition (Mak 2013, Ginsburg 2008).

**Table 2:** Meeting partners of the German Federal Constitutional Court, 1998–2019

Type of meeting partner	Meeting partner	Legal tradition	Frequency of meetings	Relative percentage
National court			**112**	**81.75**
	Constitutional Court of Austria	Civil law	8	
	Supreme Court of South Korea	Civil law	6	
	Constitutional Court of Spain	Civil law	5	
	Federal Supreme Court of Switzerland	Civil law	5	
	Conseil constitutionnel (France)	Civil law	4	
	Constitutional Court of Romania	Civil law	4	
	Constitutional Court of Slovakia	Civil law	4	
	Constitutional Court of the Czech Republic	Civil law	4	
	Constitutional Tribunal (Poland)	Civil law	4	
	Supreme Court of Israel	Mixed law	4	
	Supreme Court of the United Kingdom	Common law	4	
	Constitutional Court of Armenia	Civil law	3	
	Constitutional Court of Bulgaria	Civil law	3	
	Constitutional Court of Italy	Civil law	3	
	Constitutional Court of Lithuania	Civil law	3	
	Constitutional Court of the Russian Federation	Civil law	3	
	Constitutional Court of Turkey	Civil law	3	
	State Court of the Principality of Liechtenstein	Civil law	3	
	Supreme Court of Canada	Common law	3	
	Constitutional Court of Azerbaijan	Civil law	2	
	Constitutional Court of Belgium	Civil law	2	
	Constitutional Court of Chile	Civil law	2	
	Constitutional Court of Croatia	Civil law	2	
	Constitutional Court of Hungary	Civil law	2	
	Constitutional Court of Latvia	Civil law	2	
	Constitutional Court of Slovenia	Civil law	2	
	Constitutional Court of South Africa	Mixed law	2	
	Constitutional Court of Ukraine	Civil law	2	
	Supreme Administrative Court of Finland	Civil law	2	
	Supreme Court of Finland	Civil law	2	
	Supreme Court of Ireland	Common law	2	
	Supreme People’s Court of the People’s Republic of China	Civil law	2	
	Constitutional Court of Albania	Civil law	1	
	Constitutional Court of Bahrain	Mixed law	1	
National court	Constitutional Court of Bosnia and Herzegovina	Civil law	1	
	Constitutional Court of Georgia	Civil law	1	
	Constitutional Court of Moldova	Civil law	1	
	Federal Supreme Court of Iraq	Mixed law	1	
	Supreme Court of Appeal of South Africa	Mixed law	1	
	Supreme Court of Denmark	Civil law	1	
	Supreme Court of the United States	Common law	1	
	Tribunal Constitucional (Portugal)	Civil law	1	
Supranational court			**14**	**10.22**
	European Court of Human Rights	Mixed law	7	
	Court of Justice of the European Union	Mixed law	6	
	African Court on Human and Peoples’ Rights	Mixed law	1	
National political actor			**4**	**2.92**
	Consul General of the USA in Germany	Common law	1	
	President of Austria	Civil law	1	
	Russian Federation	Civil law	1	
	State Great Khural (Monoglia)	Civil law	1	
Supranational political actor			**3**	**2.19**
	European Commission	Mixed law	1	
	European Parliament	Mixed law	1	
	Sovereign Military Order of Malta	Mixed law	1	
National legal actor			**3**	**2.19**
	Association of Lawyers of Russia	Civil law	1	
	French Ombudsman	Civil law	1	
	Meeting of the Colombian Constitutional Jurisdiction	Civil law	1	
Supranational legal actor			**1**	**0.73**
	Annual Meeting of Latin American Judges	Mixed law	1

**Table 3:** Frequency and percentage for bilateral court meetings, 1998–2019

Legal tradition	Jurisprudential		Strategic		Total	
	Frequency	%	Frequency	%	Frequency	%
Civil law	53	74.6	46	69.7	99	72.3
Common law	6	8.5	5	7.6	11	8.0
Mixed law	12	16.9	15	22.7	27	19.7
Total	71	100	66	100	137	100
*Type of meeting partner*						
National court	65	91.5	47	71.2	112	81.8
National legal actor	1	1.4	2	3	3	2.2
National political actor	0	0	4	6.1	4	2.9
Supranational court	4	5.6	10	15.2	14	10.2
Supranational legal actor	0	0	1	1.5	1	0.7
Supranational political actor	1	1.4	2	3	3	2.2
Total	71	100	66	100	137	100

### Content analysis of the meeting reports

The jurisprudential aim implies that courts generate knowledge of foreign legal systems and national case law. The strategic aim implies that courts maintain international relations, promote the rule of law and democratic principles, and strengthen their position internationally and nationally by building credibility and authority outside their legal system (Claes and de Visser 2012; Davies 2020). Scholars argue that promoting democracy and the rule of law is more relevant for meetings with international organisations or actors from emerging countries (Kersch 2009; Garoupa and Ginsburg 2015; Dressel et al. 2018).

The directed content analysis of the meeting reports provides evidence for both considerations. Table 3 illustrates the distribution between meetings coded as strategic and jurisprudential, between the legal tradition of the FCC’s counterpart, and between the type of the meeting partner. It shows that slightly more meetings focused on jurisprudential aspects, which also holds across the legal traditions. Table 3 further reveals that bilateral meetings are more likely strategic when the FCC’s counterpart has a mixed law tradition. The table shows that 13 out of 18 meetings with supranational partners are classified as strategic meetings when focusing on the meeting partner type. This finding illustrates that strategic considerations regarding the maintenance of international relations and position reinforcement hold in the case of the FCC (Claes and de Visser 2012; Garoupa and Ginsburg 2015; Davies 2020). Moreover, it also proves that specific jurisprudential discussions are more often conducted with interlocutors that share the same legal tradition (Mak 2013).

**Fig. 3:**
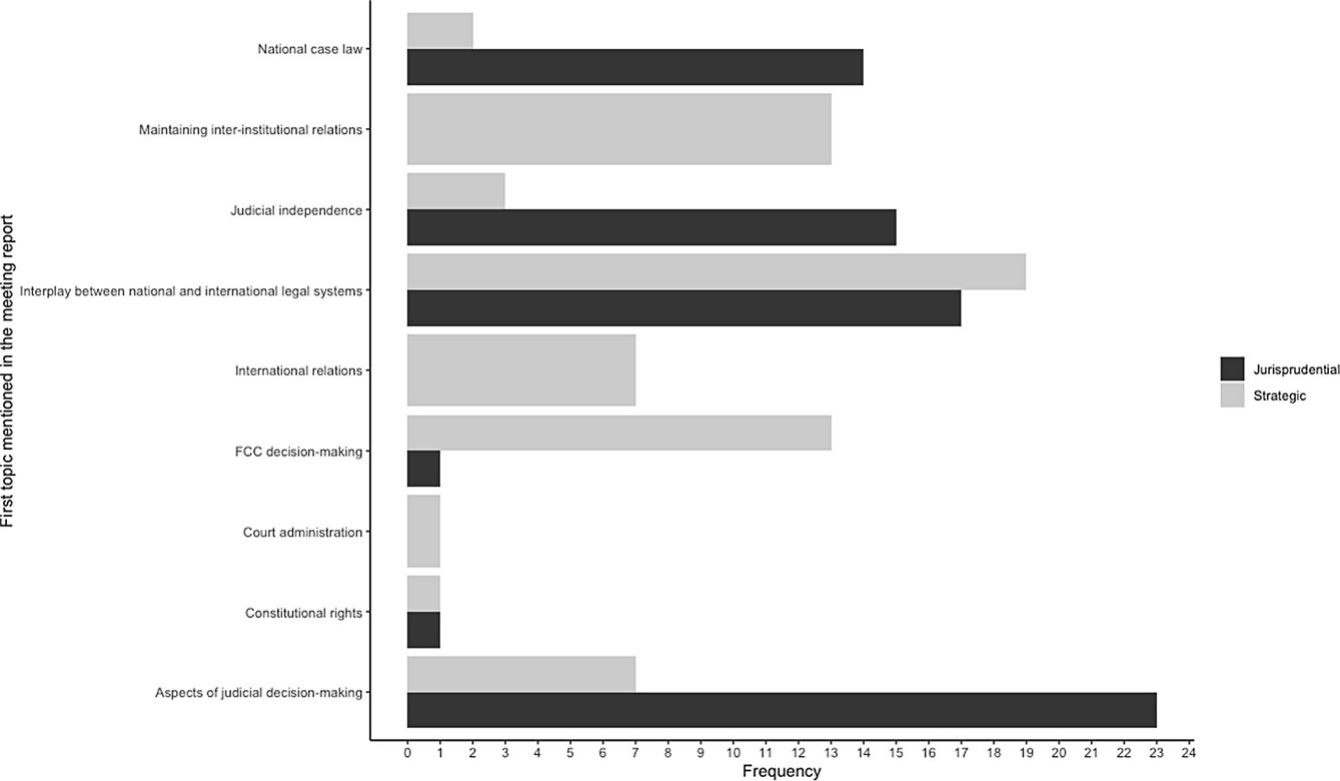
Frequency of the first issue mentioned in the meeting reports, 1998–2019

Fig. 3 illustrates the frequency distribution between meeting aims and the first topic mentioned in the FCC reports. Two aspects should be highlighted here. First, the jurisprudential aim is dominant when the meeting partner discusses national case law, judicial independence, and aspects of judicial decision-making. This finding implies that at least the FCC meetings are indeed occasions for courts “to discuss and clarify case law, and exchange views in areas of common concern and interest” (Davies 2020, p. 80). Second, the strategic aim is dominant when the meeting partner discusses the relationship between national and international legal systems, when they maintain inter-institutional relations, and when the FCC improves international relations. Hence, the FCC’s bilateral meetings could be indeed seen as “‘outreach work’ which can help courts enhance their standing among different judicial audiences” (Davies 2020, p. 80). Moreover, strategic considerations are also relevant for meetings that discuss the decision-making of the FCC. This finding is in line with Claes and Visser (2012, p. 111), who states that “courts […] could be interested in carrying over this domestic stature and position—and perhaps even exporting domestic solutions—into the international arena.”

**Fig. 4:**
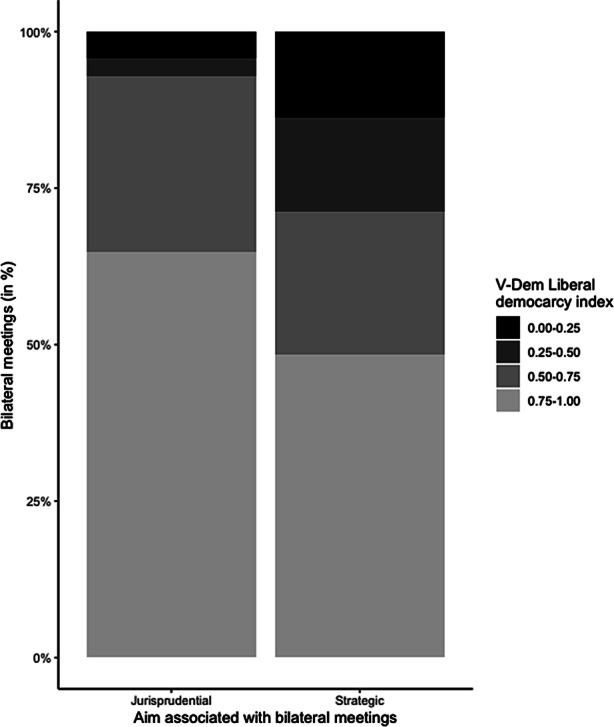
The state of liberal democracy and courts’ aims, 1998–2019

Finally, Fig. 4 considers the state of liberal democracy for each meeting partner at each meeting. The state of liberal democracy is measured with the V‑Dem liberal democracy index; it reflects the liberal and electoral principles of democracy and is measured continuously from 0 (no liberal democracy) to 1 (full liberal democracy) (Pemstein et al. 2020). The figure shows that meetings with a strategic focus are more often with partners from countries with a low and medium developed liberal democracy. In line with the liberalism theory of international relations, this finding suggests that the FCC uses its meetings to promote and foster the rule of law and, therefore, also promote democratic values. This, therefore, provides some evidence for theoretical considerations made by Garoupa and Ginsburg (2015), Slaughter (2004), and Kersch (2009).

### Semantic networks of meeting reports

**Fig. 5:**
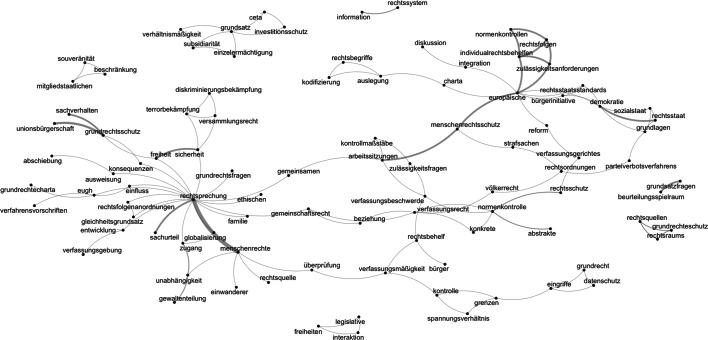
The semantic network of bilateral meeting reports by the FCC, 1998–2019

Fig. 5 illustrates the semantic network of the FCC meeting reports. Semantic networks are graphical illustrations of the proximity of word occurrences. The bigger the edges or links between the words, the more often they appear connected (Drieger 2013).

Fig. 5 shows several small word clusters like “legal sources” (*rechtsquellen*), “basic rights protection” (*grundrechteschutz*), and “legal sphere” (*rechtsraums*) (bottom right), or “legislative” (*legislative*), “freedoms” (*freiheiten*), and “interaction” (*interaktion*) (bottom-center). The figure also depicts two major world clusters: “case law” (*rechtsprechung*)—centre-left—and “european” (*europäische*)—top right. Both share a connection with the term “working meetings” (*arbeitssitungen*). Three central aspects can be derived from the network graph.

First, the strong connection between the terms “working meetings,” “constitutional complaints” (*verfassungsbeschwerde*), and “control standards” (*kontrollmaßstäbe*) show that the FCC meetings are arenas to discuss judicial-decision making practices and procedures. This finding fits with what Davies (2020) and Claes and de Visser (2012) argue to be jurisprudential or practical incentives of bilateral activities.

Second, the “case law” cluster illustrates that the discussions at those bilateral meetings centre around issues like “deciding cases on their merits” (*sachurteil*), legal “questions on basic rights” (*grundrechtsfragen*), and specific aspects of judicial decision-making like “counterterrorism” (*terrorismusbekämpfung*), “security” and “freedom” (*sicherheit; freiheit*), and the interplay between “European Union citizenship” (*unionsbürgerschaft*) and the “protection of basic rights” (*grundrechtsschutz*). Thus, bilateral meetings seem to have jurisprudential value for courts, as they offer opportunities to “improve the quality of decision-making at home […] by discussing recent case law developments, exchanging views on the application of legal principles, [and] explaining features of domestic law” (Davies 2020, p. 94). This provides evidence for the first hypothesis.

Third, the “european” cluster focuses on several topics like for example 1. “eligibility requirements” (*zulässigkeitsanforderungen*) for “individual legal remedies” (*individualrechtsbehelfen*); 2. the interplay between “democracy” (*demokratie*), the “rule of law” (*rechtsstaat*), and the “welfare state” (*sozialstaat*);* 3.* “integration” (*integration*) of European law into national law.

Another internationally-focused group of topics is displayed in the nodes between “case law” (*rechtssprechung*), “globalisation” (*globalisierung*), and “human rights” (*Menschenrechte*), which is further differentiated in judicial independence (*unabhängigkeit*), “separation of powers” (*gewaltenteilung*), and the conflicting relationship between “constitutional review” (*überprüfung, verfassungsmäßigkeit*) and the “limits” of judicial “control” (*kontrolle, grenzen*). These semantic structures show that the discussions at such meetings are of strategic value for courts, as they offer them the opportunity to “restort to judicial networking with peers […] to reinforce their domestic reputation and standing within the domestic legal order” (Claes and de Visser 2012, p. 112) and thereby “maintaining good inter-institutional relations” (Davies 2020, p. 94) with national and supranational judges, courts, and other actors. This provides evidence for the second hypothesis.

## Discussion and Conclusion

This study has asked which aims courts associate with bilateral meetings. Since previous research lacked rigorous empirical analysis and data, it has used a novel data source in international judicial relations: official meeting reports. Although reports by only one court, the German Federal Constitutional Court, were assessed here, it has been shown that several highest courts not only engage in international relations but also publish reports that share highly similar characteristics in terms of content, wording, and structure (see Table 1 & Footnote 9). Consequently, the analytical approach and results presented here can potentially travel to other cases or inspire a comparative study on judicial diplomacy efforts.

The directed content analysis results are consistent with the previous research on the aims and incentives assumed to guide courts’ activities on the international level. On the one hand, the analysis of the meeting reports provides evidence that the meetings have jurisprudential incentives for courts. Bilateral meetings arenas to discuss developments in national case law, specific procedures like constitutional complaints, and explain domestic decision-making practices and court administration features. The major contributions of these jurisprudential aims are twofold. First, courts protect domestic constitutional interests by explaining specific features of the national law and judicial decision-making (Mak 2013; Davies 2020). Second, as globalisation processes increase the probability that national judiciaries face similar problems, discussions on jurisprudential aspects contribute to the mutual understanding of different legal systems and, thus, enable judges “to see how other courts have handled similar questions” (Garoupa and Ginsburg 2015, p. 170). As such, bilateral meetings focused on jurisprudential aspects potentially increase the effectiveness of judicial decision-making.

On the other hand, the content analysis shows that courts pursue strategic aims when participating in bilateral meetings. In this context, courts discuss various issues like the relationship between national and international legal systems, the maintenance of inter-institutional relations, and the improvement of international relations. Strategic-focused meetings seem to facilitate courts’ ambitions to enhance their standing among other national and international judiciaries and protect their domestic legal order by transferring positions into the international arena (Claes and de Visser 2012; Davies 2020). Moreover, the results show that strategic aims are dominant when the German Federal Constitutional Court decision-making is mainly discussed at bilateral meetings. According to Claes and Visser (2012, p. 112, emphasis in the original), this indicates a mere political dialogue “but about what judges *want*—for instance, advocating a certain way of dealing with a legal issue.” On a final note, the results also provide evidence that first, transjudicial communication happens more often between discussion partner that shares the same legal tradition, particularly when discussing jurisprudential aspects (Mak 2013; Law 2015) and second, in bilateral meetings with interlocutors from emerging democracies, the strategic aims come more into focus, which suggests that the promotion of the rule of law and support for democracy are the main motivations here (Slaughter 2004; Kersch 2009).

The semantic structures analysis within the meeting reports provides additional evidence for these findings. Bilateral meetings are found to be dominated mainly by two clusters: case law and issues regarding the European Union. These dominant clusters are further associated with supporting clusters regarding specific decision-making procedures like constitutional complaints, globalisation, human rights, judicial independence, and judicial review. Overall, this study finds empirical evidence for existing scholarship on judicial diplomacy and thereby sheds light on the informal dimension of judicial politics (see also Dressel et al. 2018). Hence, it presents both an analytical and data-related contribution to this literature.

Bilateral court meetings are opportunities for courts to increase mutual knowledge and understanding. The exchanges considered here were evenly concerned with procedural issues like judicial administration and substantial issues like counterterrorism or securing basic rights. Moving forward, scholars should overcome this major drawback of the study by applying comparative methods or automated text classification algorithms. At the same time, future research could also use this study as a starting point to assess how bilateral court meetings benefit judicial dialogue through mutual decision citation. Other aspects that this study has not adequately discussed are some important properties of the meeting reports. For example, it is unclear whether the reports emphasise some aspects over others or whether they are influenced by current political affairs, crises, and recent developments. These aspects need to be discussed in future scholarly work.

David Law (2015, p. 1024) argues that the “globalisation of constitutional law is characterised not only by the emergence of generic or universal elements but also by the persistence of distinct constitutional families.” One consequence he draws is that prestigious national courts, especially the US Supreme Court and the German Federal Constitutional Court, use judicial diplomacy to steadily increase their influence on other national judiciaries (Law 2015, p. 1024–1025). Does judicial diplomacy strengthen distinct global constitutional families’ persistence, as courts more likely meet with courts that share the same legal tradition? Why is there so little engagement of the German court with courts from Africa and does the colonial heritage influence a court’s diplomatic efforts? How much do the German court’s diplomatic efforts differ from other influential courts such as the French *Conseil constitutionnel*? Future studies could collect the available information and reports, which seem to share structural commonalities which are easy to compare, on international judicial relations from several national highest courts (see Table 1) and use the analytical approach presented here to enable a comparative perspective on judicial efforts into international between-bench relations. Further, Figs. 3 and 4 have shown that the meeting topics differentiate according to the political context of the interlocutor. Future research should use this result to assess whether there was also a difference in the behaviour of the judges when meetings took place in a court’s country or the interlocutor’s country. Moreover, this study has shown that the theoretically claimed strategic and jurisprudential aims hold empirically. In order to overcome the reliability and validity problems of content analysis, future studies could use the coding categories presented here and employ quantitative text analysis methods that require predefined categories such as semi-supervised text classification based on seed dictionaries (Watanabe and Zhou 2020) or latent semantic scaling (Watanabe 2020).

Finally, international judicial relations expand national highest courts’ functions as guardians and developers of the law. Most importantly, this encompasses “a proliferation of foreign legal norms, which have a binding or inspiring status in the decision-making of the highest courts” (Mak 2013, p. 137). In this regard, several scholars have assumed that the citation of foreign court decisions and other means of cross-fertilisation is a product of judicial diplomacy (Slaughter 2004; Meierheinrich 2009; Law and Chang 2011). However, previous research has not yet gone beyond mere description (Meierheinrich 2009). This study has demonstrated the advantages of an empirical analysis of text data on international judicial relations. Moving further by taking up a comparative perspective, future research could use data on bilateral meetings in order to understand when and how courts discuss recent case law developments and link this to the research on citation networks and the citation of foreign precedents (e.g., Dyevre 2010; Dyevre *et al.* 2019; Fowler *et al.* 2007). Thereby, research on bilateral court meetings can provide a missing link in understanding the global judicial architecture.

## Supplementary Information

Supplementary Appendix

## Footnotes

1 Legal globalisation describes processes that aim to create global legal standards and international regulations, which the nation-states must comply with if they want to be economically successful. Benvenisti and Downs (2009, p. 63) even state that the “[l]aw increasingly has replaces diplomacy as the medium in which states interact and act collectively.”.

2 Bilateral meetings are understood as visits by or to the Court to or from foreign courts or foreign political actors. For example, a delegation from the Kremlin, the US consul general in Germany, or a Mongolian Parliament delegation. Although these non-judicial meetings are relatively rare, they imply that the FCC seems to be an autonomous diplomatic actor in German international relations. This assumption can be further strengthened by the fact that several heads of state and government have regularly invited its judges in the context of a visit to a foreign court. These aspects will be briefly further discussed in the analysis.

3 Meierheinrich (2009) argues that Slaughter’s threefold conceptualisation is blurred and imprecise, as the concepts encompass a relatively broad range from bilateral meetings to law enforcement actors like *Interpol*.

4 Similar considerations can be found in several other studies (Slaughter 2004; Benvenisti and Downs 2009; Claes and de Visser 2012; Mak 2013; Garoupa and Ginsburg 2015).

5 Legal characteristics are also reflected in the judicial systems. The judiciary in common law systems has an equal status to the executive and the legislature. The process of judicial review is *diffused* and *concrete*. Every court can review laws and acts by authorities while the Supreme Court presents the final authority (*diffuse*). An actual case must provide for a legal dispute (*concrete*), and judicial review is limited to laws in force (*a posteriori* review). Every person who is engaged in a real case can initiate a dispute settlement. The judiciary in civil law systems is separated into a judicial branch for cases concerning ordinary laws with various courts and a judicial branch for cases concerning constitutional law with the Constitutional Court as a separate institution. Thus, the civil law system is a *specialised* system of judicial review. The review of laws and disputes can be concrete and *abstract* in the absence of a real case. Judicial review can be posteriori and *a priori* when a law in question is not yet in force. Plaintiffs are executive and legislative actors, political and civil organisations, companies, as well as individual citizens (see, e.g., Epstein et al. 2001).

6 On the contrary, the German court is known for being open in its adjudication process to consider various (national and international) legal sources (Law 2015).

7 The first meeting report since 2019 was published in October 2020, covering a meeting with the Court of Justice of the European Union.

8 Although the meeting reports are relatively short and sparse documents, they provide a suitable amount of information for the methods presented in the following. Modern computational text analysis methods have grown powerful to assess short texts by, for example, treating every individual sentence or even individual words as documents, which is a common strategy to analyse social media posts (Watanabe 2020).

9 A comparison of the meeting reports of the FCC with those of the constitutional courts of France (listed in its annual reports), Austria, and Slovenia (both courts publish, like the FCC, press releases regarding their meetings) reveals similar patterns in terms of content, wording, and structure. All three compared courts publish reports which contain very brief descriptions of the meeting content alongside the participating judges and the type of the meeting (e.g. workshop). This comparison illustrates that meeting reports seem to share common characteristics and systematics.

10 The preprocessing and the semantic network analysis are done in *R* with the *quanteda *package (Benoit et al. 2018).

11 For 2019, the annual reports of the Conseil constitutionnel (https://www.conseil-constitutionnel.fr/sites/default/files/2019-10/2019rapdf_bd_en.pdf, accessed November 16, 2021) and the Norwegian Supreme Court (https://www.domstol.no/en/Enkelt-domstol/supremecourt/annual-report/2019/, accessed November 5, 2020) report seven bilateral meetings with international actors.
