# Supplementary material for: Judicial diplomacy of the German Federal Constitutional Court: bilateral court meetings as a novel data source to assess transnational communication of constitutional courts
Source: Z Vgl Polit. 2021 Dec 20;15(3):295–323. doi: 10.1007/s12286-021-00499-0 (PMC8686088; doi:10.1007/s12286-021-00499-0)

**Supplementary Appendix**

The appendix for this paper contains three parts. Appendix A shows examples of the meeting reports published by the German Federal Constitutional Court. Appendix B is concerned with the coding rules for the directed content analysis and shows the coding categories, their definitions, and examples for each category.

***Appendix A: Examples of meeting reports published by the German Federal Constitutional Court***

| **Example 1:**  **Meeting location: German Federal Constitutional Court, Karlsruhe**  **Meeting date: 08.05.2007**  **Dialogue partner of the FCC: Delegation of judges from the African Court on Human and Peoples' Rights**  Richter des Afrikanischen Gerichtshofes für Menschenrechte besuchen das Bundesverfassungsgericht  Pressemitteilung Nr. 51/2007 vom 7. Mai 2007  Am Dienstag, den 8. Mai 2007, besucht eine Delegation von Richtern des Afrikanischen Gerichtshofes für Menschenrechte (AGHMR) das Bundesverfassungsgericht. Teilnehmende Richterinnen und Richter der Delegation sind:  Herr Gerad Niyungeko, Burundi, Präsident des AGHMR  Herr Tounty Guindo, Mali, Vizepräsident  Frau Sophia A.B. Akuffo, Ghana  Herr El Hadji Guisse, Senegal  Frau Kelello Justina Mafoso-Guni, Lesotho  Herr Jean Mutsini, Ruanda  Herr Bernard Makgabo Ngoepe, Südafrika  Herr Fatsah Ouguergouz, Algerien  Herr Jean Emile Somda, Burkina Faso  Der Afrikanische Menschenrechtsgerichtshof ist eine neue Einrichtung der afrikanischen Staaten und soll 2008 seine Arbeit aufnehmen. Seine Aufgabe besteht in der Überprüfung der Einhaltung der Menschenrechte in den afrikanischen Staaten. Er besteht aus elf Richterinnen und Richtern und spiegelt in seiner Zusammensetzung die verschiedenen Regionen Afrikas wider. Zur Vorbereitung auf ihre Aufgaben werden die Richter des Gerichtshofes zusammen mit dem Präsidenten des Bundesverfassungsgerichts, Herrn Prof. Dr. Dres. h.c. Hans-Jürgen Papier, und weiteren Richterinnen und Richtern des Bundesverfassungsgerichts Fachgespräche zu verschiedenen Fragen der Verfassungsgerichtsbarkeit führen.  https://www.bundesverfassungsgericht.de/SharedDocs/Pressemitteilungen/DE/2007/bvg07-051.html |
| --- |
| **Example 2:**  **Meeting location: German Federal Constitutional Court, Karlsruhe**  **Meeting date: 24.05.-26.05 2000**  **Dialogue partner of the FCC: Delegation of judges from the Constitutional Court of Austria**  Besuch einer Delegation des Österreichischen Verfassungsgerichts  Pressemitteilung Nr. 68/2000 vom 23. Mai 2000  Vom 24. bis zum 26. Mai 2000 wird eine Delegation des Österreichischen Verfassungsgerichtshofs das BVerfG besuchen. Mit diesem Besuch wird eine langjährige Tradition des Gedanken- und Erfahrungsaustauschs fortgesetzt. In einem Rhythmus von ca. 3 Jahren pflegen sich die Gerichte abwechselnd in Karlsruhe oder Wien zu Gesprächen über die Entwicklung ihrer Rechtsprechung zu treffen.  Die insgesamt acht richterliche Mitglieder umfassende Delegation wird vom Präsidenten des Gerichts, Prof. Dr. Ludwig Adamovich, und dem Vizepräsidenten, Prof. Dr. Karl Korinek, geleitet.  Die Richter und Richterinnen aus Wien werden Fachgespräche mit Präsidentin Prof. Dr. Limbach, Vizepräsident Prof. Dr. Hans-Jürgen Papier und weiteren Richterinnen und Richtern aus beiden Senaten des BVerfG führen. Dabei soll es u.a. um Fragen einer Grundrechts-Charta für die Europäische Union, die Reform des Österreichischen Verfassungsgerichtes sowie den vorläufigen Rechtsschutz gehen.  Darüber hinaus werden die Gäste die Universität Heidelberg besuchen und anschließend von der Oberbürgermeisterin der Stadt, Frau Beate Weber, empfangen.  Karlsruhe, den 23. Mai 2000  https://www.bundesverfassungsgericht.de/SharedDocs/Pressemitteilungen/DE/2000/bvg00-068.html |
| **Example 3:**  **Meeting location: Supreme Court of Israel, Jerusalem**  **Meeting date: 15.11.2019**  Besuch des Bundesverfassungsgerichts beim Obersten Gerichtshof des Staates Israel  Pressemitteilung Nr. 80/2019 vom 15. November 2019  Eine Delegation des Bundesverfassungsgerichts unter Leitung des Präsidenten Prof. Dr. Dres. h. c. Andreas Voßkuhle und des Vizepräsidenten Prof. Dr. Stephan Harbarth, LL.M., besuchte vom 12. bis 15. November 2019 den Obersten Gerichtshof des Staates Israel. Die Delegation wurde dabei von der Präsidentin Esther Hayut und weiteren Mitgliedern des Obersten Gerichtshofs empfangen. Neben der Meinungsfreiheit in Zeiten des Internets und der Unabhängigkeit der Gerichte, waren die Menschenrechte insbesondere im Hinblick auf Einwanderer und Asylbewerber Thema der Fachgespräche. Darüber hinaus dienten die Gespräche dem Austausch über die Rechtsprechung beider Gerichte.  https://www.bundesverfassungsgericht.de/SharedDocs/Pressemitteilungen/DE/2019/bvg19-080.html |
| **Example 4:**  **Meeting location: Constitutional Court of Hungary, Budapest**  **Meeting date: 23.09-25.09.2013**  Besuch des Bundesverfassungsgerichts beim ungarischen Verfassungsgericht  Pressemitteilung Nr. 58/2013 vom 25. September 2013  Das Bundesverfassungsgericht hat das Verfassungsgericht der Republik Ungarn auf dortige Einladung vom 23. bis 25. September 2013 besucht. Der Delegation gehörten Präsident Prof. Dr. Andreas Voßkuhle, Vizepräsident Prof. Dr. Ferdinand Kirchhof sowie weitere Richterinnen und Richter des Bundesverfassungsgerichts an. In den Fachgesprächen tauschten sich die Mitglieder der beiden Gerichte unter anderem über das Verhältnis der nationalen Verfassungen zum Recht der Europäischen Union aus. Weitere Themen waren die Zulässigkeitsvoraussetzungen im Verfassungsbeschwerdeverfahren sowie wichtige aktuelle Entscheidungen der beiden Gerichte.  https://www.bundesverfassungsgericht.de/SharedDocs/Pressemitteilungen/DE/2013/bvg13-058.html |
| **Example 5:**  **Meeting location: German Federal Constitutional Court, Karlsruhe**  **Meeting date: 29.10.-30.10 2015**  **Dialogue partner of the FCC: Delegation of judges from the Constitutional Court of Korea**  Besuch des Verfassungsgerichts der Republik Korea beim Bundesverfassungsgericht  Pressemitteilung Nr. 79/2015 vom 30. Oktober 2015  Am 29. und 30. Oktober 2015 besuchte eine Delegation des Verfassungsgerichts der Republik Korea unter Leitung seines Präsidenten Park Han-Chul das Bundesverfassungsgericht. Die Delegation wurde von Präsident Prof. Dr. Andreas Voßkuhle, Vizepräsident Prof. Dr. Ferdinand Kirchhof sowie Richterinnen und Richtern des Bundesverfassungsgerichts empfangen. Die Fachgespräche beleuchteten unter anderem die rechtlichen Grundlagen des Parteiverbotsverfahrens in den beiden Rechtsordnungen. Zudem fand ein Erfahrungsaustausch über den Schutz sozialer Grundrechte statt.  https://www.bundesverfassungsgericht.de/SharedDocs/Pressemitteilungen/DE/2015/bvg15-079.html |
| **Example 6:**  **Meeting location: Constitutional Tribunal of Poland, Warsaw**  **Meeting date: 25.05-26.05.2004**  Besuch des Verfassungsgerichtshofs der Republik Polen  Pressemitteilung Nr. 54/2004 vom 25. Mai 2004  Auf Einladung des Präsidenten des Verfassungsgerichtshofs der Republik Polen, Herrn Prof. Marek Safjan, besucht der Präsident des Bundesverfassungsgerichts, Prof. Dr. Hans-Jürgen Papier, am 25. und 26. Mai 2004 den Verfassungsgerichtshof der Republik Polen in Warschau.  Im Vordergrund des Besuchs stehen Fach- und Seminargespräche zwischen den Richtern des Verfassungsgerichtshofs der Republik Polen und dem Präsidenten des Bundesverfassungsgerichts. Der Präsident des Bundesverfassungsgerichts wird in Warschau vor den Mitgliedern des Verfassungsgerichtshofs über die Themen "Der Schutz von Ehe und Familie in der Rechtsprechung des Bundesverfassungsgerichts" und "Probleme der Durchsetzung verfassungsgerichtlicher Entscheidungen" sprechen.  Der Präsident des Bundesverfassungsgerichts wird außerdem vom Präsidenten der Republik Polen, Herrn Aleksander Kwasniewski, empfangen werden.  Karlsruhe, den 25. Mai 2004  https://www.bundesverfassungsgericht.de/SharedDocs/Pressemitteilungen/DE/2004/bvg04-054.html |

***Appendix B: Coding rules for the directed content analysis: categories, definitions, examples***


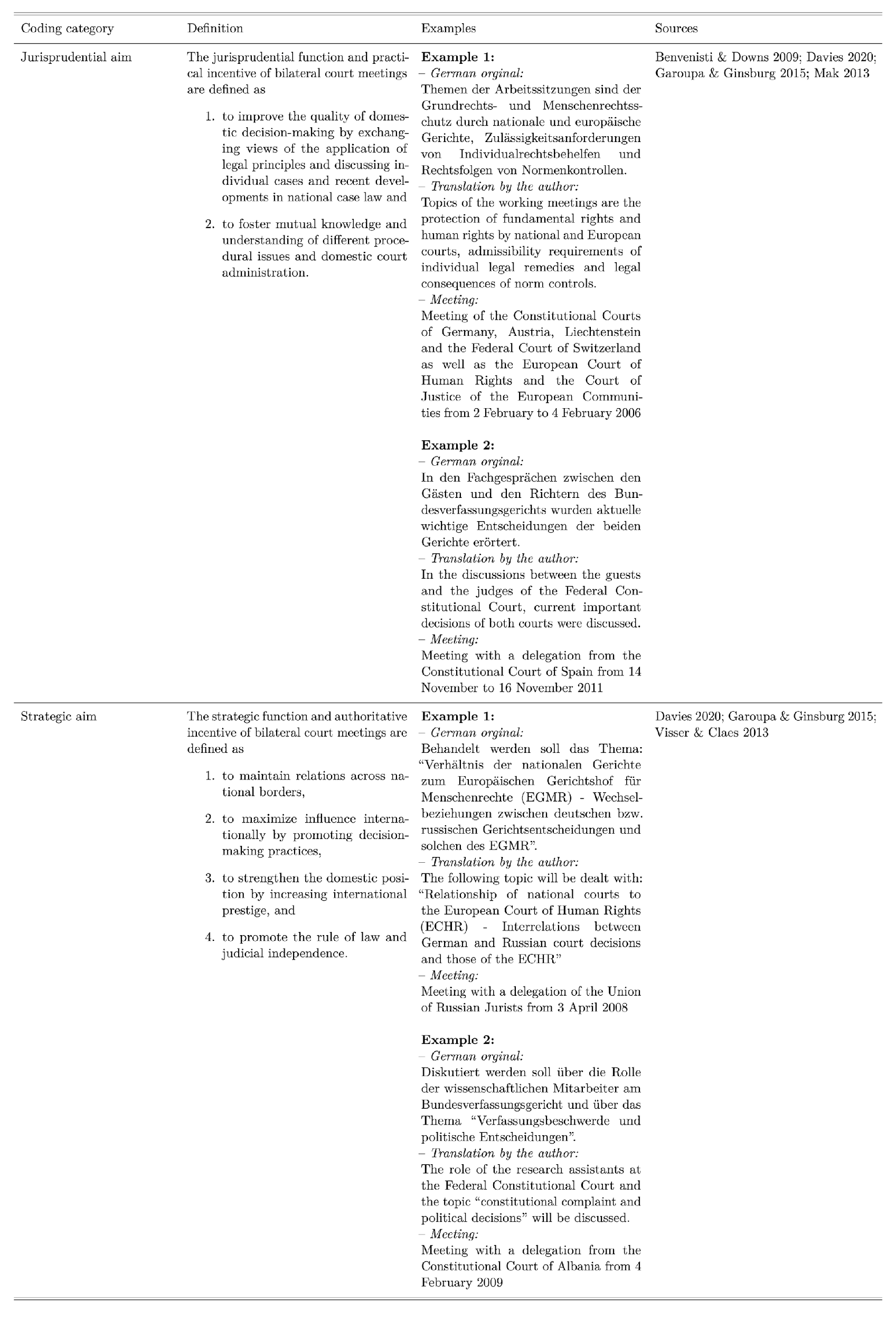
***Appendix C: Description and examples of the issues discussed at the bilateral meetings***


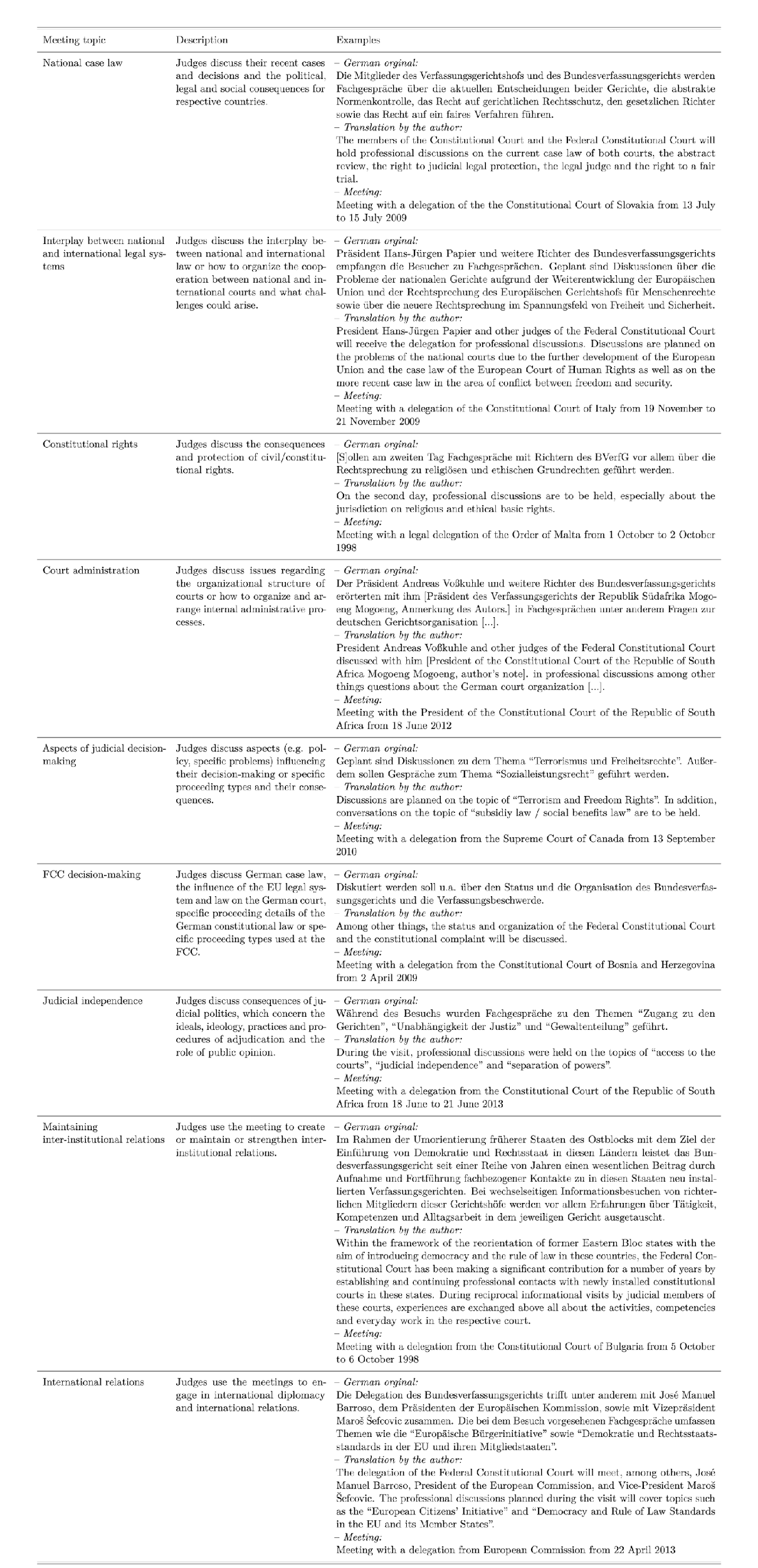

Supplement: Supplementary file 1 — Supplementary Appendix [file 12286_2021_499_MOESM1_ESM.docx]
